# Supplementary figures and images for: Safety, efficacy and airway complications of the flexible laryngeal mask airway in functional endoscopic sinus surgery: A retrospective study of 6661 patients
Source: PLoS One. 2021 Feb 4;16(2):e0245521. doi: 10.1371/journal.pone.0245521 (PMC7861430; doi:10.1371/journal.pone.0245521)

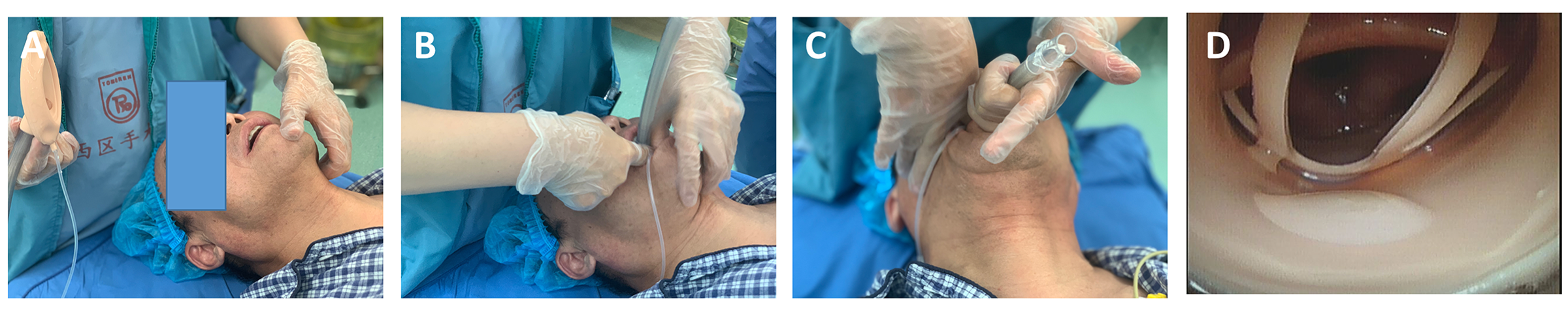

Supplement: S1 Fig — (A) Opening the oral cavity; (B) Placing the laryngeal mask into the oral cavity; (C) Lacing the laryngeal mask into the pharyngeal cavity; (D) FOB view. (TIF) [file pone.0245521.s001.tif]
