# Supplementary material for: Safety, efficacy and airway complications of the flexible laryngeal mask airway in functional endoscopic sinus surgery: A retrospective study of 6661 patients
Source: PLoS One. 2021 Feb 4;16(2):e0245521. doi: 10.1371/journal.pone.0245521 (PMC7861430; doi:10.1371/journal.pone.0245521)
Supplement: Attachment — Submitted filename: Reviewers comments.docx [file pone.0245521.s002.docx]

Summary: the manuscript claims to report safety, efficacy and airway complications of the flexible laryngeal mask airway in functional endoscopic sinus surgery as retrospective study. The strength of the study is author’s efforts in conducting a large retrospective study. We commend them to perform this study in a single centre. The weakness is the flaw in the methodology and flow of language and content. It needs a major revision in various areas. The main points are highlighted below.

Abstract:

In the objective section the primary aim is not mentioned but states safety and efficiency.

What is the main objective? The authors should clarify it.

In methods though Primary outcome was the success rate, the secondary aim does not specify clinical factors which are related to the failure. Authors needs to be clarify it to avoid confusion

Conclusion: Instead of reporting the results of primary outcome, authors conclude on FLMA safety and efficacy. In the second sentence the author’s mention that the intra operative failure is uncommon. But failed to the failure rate. I don’t know what the latter half of the sentence means: “detected quickly and handled immediately”. The data or text in the results doesn’t support it.

Methods: What is EEG?

Methodology is inadequate. Inclusion criteria is only the time period of data collected but doesn’t give other details. Inclusion or exclusion criteria doesn’t mention on patients who had FESS with ETT.

In results in line 145 the some details were given. Example: 129 (1.9%) were scheduled for ETT .

Then line 88: what I can understand is the departmental standardised GA options. It is not clear whether it’s their departmental protocol. If it is, whether it was applied in all cases by all the anaesthetists. Doesn’t mention on non-availability of data in exclusion criteria.

Line 104: can’t understand what authors want to say. What is lead around the patient neck?

Results: Line 144,149,-153: 6661 patients were initiated and final success of FLMA was 6512.

The number do not add up to the figure of 6512. Based on the failures (89+60+14+46=209) it should be 6452. Please check.

Table 1 only one p value (0.008) was given for all LMAs. Is it different for all sizes?

Similarly for ASA grade, does one p value <0.001 applies to all grades?

Table 3 the total respiratory adverse events given as (n) 56 nerve. Adverse events under this subheading are they different to above total? If it is 56 then events mentioned do not add up. The subheading on tissue damage adverse events are immediate and delayed complications, suggest change of wording.

Discussion: under safety and efficacy: did all cases were compliant in maintaining the OLP and PIP difference of 5H20. Line 226 mentions inadequate relaxation, whether neuromuscular monitoring was carried out?

Line 227- 233: Author’s mentions sometimes reducing tidal volume and increasing respiratory frequency were required. Is it author’s opinion on its management or speculation what occurred?

The line 246: previous study is on prevalence, risk factors, and management of asthma in China: a national cross-sectional study. I don’t understand the relevance here as this is FESS study assessing the safety and efficacy of the airway.

Awake FLMA: what’s the relevance in your retrospective study? Has the authors conducted any such procedures?

Line 260 and 261 contradicts. If not documented in the medical chart, then is it author’s opinion?

In results there were two patients had laryngospasm had NPPE. Describing their management in discussion may not be necessary?

Line 281: says no life threatening damage, but authors mentioned 5 such cases. It contradicts the previous statement.

Limitations paragraph is mixed with limits and many improvement suggestions for the future. It’s confusing to the readers.

Line 301: ranks top: superlative may be avoided

Conclusion: please refer to the comment in the abstract.

References: adequate
