# Supplementary material for: Safety, efficacy and airway complications of the flexible laryngeal mask airway in functional endoscopic sinus surgery: A retrospective study of 6661 patients
Source: PLoS One. 2021 Feb 4;16(2):e0245521. doi: 10.1371/journal.pone.0245521 (PMC7861430; doi:10.1371/journal.pone.0245521)
Supplement: Attachment — Submitted filename: 12-19-Response to Reviewers.docx [file pone.0245521.s004.docx]

Dear Editor,

I greatly appreciate you and the reviewers for all the comments, critiques and efforts to help optimize our manuscript. Both the editor and reviewers suggest that our manuscript requires a thorough grammatical and spelling review by a native English speaker to improve its readability. We asked American Journal Experts (AJE) to help us. Our manuscript has been edited by the highly qualified native English speaking editors at American Journal Experts (AJE), and the AJE certification has been uploaded a file.

During the first communication, we think that we misunderstand the query No.8 of Reviewer #2, and we’ve made a better revision in this manuscript. We are happy to respond to the comments.

Our point-by-point responses are listed below.

**Reviewer #2:**

**1 Table 3 the total** **respiratory adverse events given as (n) 56 nerve. Adverse events under this subheading are they different to above total? If it is 56 then events mentioned do not add up. The subheading on tissue damage adverse events are immediate and delayed complications, suggest change of wording.**

**The query no 8 is not answered. Table 3 the total** **respiratory adverse events given as (n) 56 The events given do not add up to 56.**

In the original manuscript, we thought the delayed complications such as sore throat and soft tissue injuries were respiratory adverse events. We think it is unappropriated and we’ve made a better Table 3 for respiratory adverse events, and the number adds up to 56.

**2. Many language or grammatical mistakes, which I cannot go thru each one. Example given below. If FLMA ventilation failed after three insertion attempts during the induction period, ETT was immediately performed**

**Line 150 May be reworded as: If FLMA ventilation failed after three insertion attempts during the induction period, patient was immediately intubated with ETT

Line 156 ETT was implemented
Suggest rewording: intubated with ETT**

Thank you for pointing out our language and grammatical mistakes. We should pay more attention on the fluency of our manuscript. We’ve asked  the highly qualified native English speaking editors at American Journal Experts (AJE) for editing services, including grammar, phrasing and punctuations. We've revised many language and grammatical mistakes.
